# Supplementary material for: Patterns of Intron Gain and Loss in Fungi
Source: PLoS Biol. 2004 Nov 30;2(12):e422. doi: 10.1371/journal.pbio.0020422 (PMC532390; doi:10.1371/journal.pbio.0020422)
Supplement: Table S1 — Also available at http://genes.mit.edu/NielsenEtAl/. (4.3 MB ZIP). [file pbio.0020422.st001.zip › NielsenEtAl/html/1267.html]

AN1387.1.NCU03517.1.MG06728.1.FG06033.1


```
 CLUSTAL W (1.82) Multiple Sequence Alignments - Introns Inserted


Sequence 1: NCU03517.1	372 aa
Sequence 2: FG06033.1	318 aa
Sequence 3: AN1387.1	306 aa
Sequence 4: MG06728.1	331 aa
Alignment Length: 377 aa
Number Identitical Residues: 123 aa
Alignment Score (without introns) 6605


MG06728.1 	----~-----------M0SKQY---------LTVHT------IDNA1HQSQIFALATTPN
NCU03517.1	MADN2CLSPTPEPLLSF~SKQLPSRDNFLSQTTLHSPRVWTDSDNA1HPTEIFSLAPTPN
FG06033.1 	----~-----------M0SKQY---------LTTHT------VDNA1HITDIFSIATTPK
AN1387.1  	----~----------MN0SKQY---------LSYGS------ADNT1HPTDIFALAVTEK
          	                  ***           :  :       **: * ::**::* * :

MG06728.1 	GVFSASGGEAIKFHSAATDATGASSFSSSDIPKAHRSGCHHLAAGAGGPGKVLASAGFGH
NCU03517.1	ALISASGSSHLLIHSTTTS---PTFPLTQTLSNAHRLGCHHITTARGGPGNTFVSVGFGG
FG06033.1 	AVISGSGSSTLHIHDTTD----PSFPLKQSISDAHKLGCHHVCASRNG--NVAASAGFGG
AN1387.1  	QILSASGSNALQVHSTTN----PDFPLVQTL-EAHKAGCHHVVTDAKG--SRAVSVGFGG
          	 ::*.**.. : .*.::     .     . : .**: ****: :   *  .  .*.*** 

MG06728.1 	EVKVWVQQEGSGEWKEHGTVSPWGSGPSAAKVWAVALNSDEQFLACSSEAGKVAVWDIPA
NCU03517.1	DTKVWHRNPETDQWSLYWTLPTPDNKKEKGDVWAVALSADEGYLAATTSDGRIHVWDVLG
FG06033.1 	EVKTWKVNNDTGEWSLSGEIAGASTKP--GEAWALALSEDGSYLATTTNDGRINVWDIVD
AN1387.1  	EVVIWESHEGT--WSKTKDVVLA-------DIWAVALSADGQYLAGTTQDGHVKVWDMNA
          	:.  *  :  :  *.    :          . **:**. *  :** ::. *:: ***:  

MG06728.1 	G--RAVHLYETAS----------EGQGSFGMSVDI~SPDGRLTASGHQNGTVYVFNNDSG
NCU03517.1	--KAKIQTYETGASAGGGGGHDSVGTGSFAMAVDL~SRDGKFTASGHENGGVYVFNNDAG
FG06033.1 	EKKPKIREYETG----------SAGSGSFGMSVDL~SRDGKFTASGHQNGSVYIFNNDTG
AN1387.1  	N-EEEIRDHETK--------------GSFGTCIDL0SPDGRFIASGHENGSVYIFSTETG
          	  .  :: :**               ***. .:*: * **:: ****:** **:*..::*

MG06728.1 	RLVYSLTG1LSKPIRSVKFSPGGTRLATAGDAGIIAIYDTQNGEHVSNLSPSGPSGPWIM
NCU03517.1	RMVYSLSG1LAKPIRAVAFSPGCKRLAAAGNAGIIAIYDMQHGEHVANLSATGTRPAWIT
FG06033.1 	RVLYSLSG1LAKPVRSVAFSPGNTRLAAAGDAGIIAIYDMKHGEHIANLTG---HSSWIT
AN1387.1  	RMPFSLSG1LVKPVRSVAFSPGGKFLAAAGDSRVIVLYDTTSGEQVASLTG---HAAWIL
          	*: :**:* * **:*:* **** . **:**:: :*.:**   **:::.*:      .** 

MG06728.1 	SLDWSHTGEYLLSG~SFDGRVRVWSIEAATCVATHSESDETIWAVKWLPKP----AGNPR
NCU03517.1	SIDWNDTGEYLLSG~SLDGKVRVWDVARGVCVATHSETESALWCVRWLPKSEKVLAPGMG
FG06033.1 	SLDWSDTGEYLLSG~SMDGKVKVWSIERTTCVATHSETDKALWSVKWLPKT--------V
AN1387.1  	SLSWSNTGEYLLSG2SFDGKVKVWSIDTRNCVATHSETERAIWSVIWLPKI--------G
          	*:.*..******** *:**:*:**.:    *******:: ::*.* ****          

MG06728.1 	AAESFCAAGARGSITFYREASGS
NCU03517.1	KGEMFVAAGANRSLTFYREATGS
FG06033.1 	RSEMFCTAGANRSLSFYREATGG
AN1387.1  	KSEGFATAGANRSISFYREATGG
          	 .* * :***. *::*****:*.
```
